# Diabetic Foot Syndrome as a Possible Cardiovascular Marker in Diabetic Patients

**DOI:** 10.1155/2015/268390

**Published:** 2015-03-26

**Authors:** Antonino Tuttolomondo, Carlo Maida, Antonio Pinto

**Affiliations:** Dipartimento Biomedico di Medicina Interna e Specialistica, U.O.C di Medicina Interna e Cardioangiologia, Università degli Studi di Palermo, Piazza delle Cliniche, No. 2, 90127 Palermo, Italy

## Abstract

Diabetic foot ulcerations have been extensively reported as vascular complications of diabetes mellitus associated with a high degree of morbidity and mortality; in fact, some authors showed a higher prevalence of major, previous and new-onset, cardiovascular, and cerebrovascular events in diabetic patients with foot ulcers than in those without these complications. This is consistent with the fact that in diabetes there is a complex interplay of several variables with inflammatory metabolic disorders and their effect on the cardiovascular system that could explain previous reports of high morbidity and mortality rates in diabetic patients with amputations. Involvement of inflammatory markers such as IL-6 plasma levels and resistin in diabetic subjects confirmed the pathogenetic issue of the “adipovascular” axis that may contribute to cardiovascular risk in patients with type 2 diabetes. In patients with diabetic foot, this “adipovascular axis” expression in lower plasma levels of adiponectin and higher plasma levels of IL-6 could be linked to foot ulcers pathogenesis by microvascular and inflammatory mechanisms. The purpose of this review is to focus on the immune inflammatory features of DFS and its possible role as a marker of cardiovascular risk in diabetes patients.

## 1. Definition of Diabetic Foot Syndrome

Complications of foot ulcers are the major cause of hospitalization and amputation in diabetic patients and lead to significant health care costs as evidenced by the fact that 20–40% of health care resources are spent on diabetes-related diabetic foot [1, 2]. Diabetic foot syndrome (DFS) is defined, according to the World health Organization, as “ulceration of the foot (*distally from the ankle and including the ankle*) associated with neuropathy and different grades of ischemia and infection” [3]. It represents a serious long-term complication of diabetes mellitus leading to amputations, disability, and reduced quality of life.

## 2. Epidemiology of Diabetic Foot Syndrome

Approximately 82000 lower extremity amputations directly related to diabetes are performed in the USA annually [4]. Of these amputations, the majority (80%) have been preceded by foot ulceration [5]. Foot ulceration is the most common single precursor to lower extremity amputation among diabetics [6]. The presence of foot ulceration is considered an important risk for morbidity, mortality, and disability as supported by the fact that about 80% of nontraumatic amputations are caused by the presence of diabetes and 85% of these amputations are preceded by a foot ulceration [7]. An estimated 15% of patients with diabetes will develop a lower extremity ulcer during the course of their disease [8].

Several population-based studies indicate a 0,5% to 3% annual cumulative incidence of diabetic foot ulcers [9]. The prevalence of foot ulcers reported for a variety of populations ranges from 2% to 10% [10]. In a retrospective US cohort study of 8.905 patients with type 1 and type 2 diabetes, the incidence of DFS was 5,8% over an observational period of 3 years [9]. In 15,6% of patients with DFS, lower limb amputations were necessary and survival was significantly shortened and the cost for 40–65-year-old male with new foot ulcers was $27987 for the two years after diagnosis. Pathogenesis of diabetic foot ulcers is complex and multifactorial and it is well known that these lesions rarely result from a single pathology. Several causes work together leading to foot ulceration in diabetic patients. The most common components of this detrimental pathway which lead to foot ulcerations include peripheral neuropathy, foot deformity, abnormal foot pressures, limited joint mobility, external trauma, peripheral vascular disease, and peripheral edema (see Figures 5 and 6). A frequently diabetes-related complication is neuropathy representing the most important contributory cause in the pathway to ulceration. Diabetic peripheral neuropathy (DPN) is an impairment of normal activities of the nerves throughout the body and can alter autonomic, motor, and sensory functions [11]. In sensory neuropathy, the lack of protective sensation makes the foot vulnerable to unattended minor injuries caused by an excess of pressure and mechanical or thermal injury. According to an important prospective multicenter study, sensory neuropathy was the most frequent component in the causal sequence to ulceration in diabetic patients [12]. Other forms of neuropathy may also play a role in foot ulceration. Motor neuropathy alters the biomechanics and, gradually, the foot anatomy causing foot deformities, limited joint mobility, and altered loading of the foot. These disarrangements may also alter the distribution of forces during walking and cause reactive thickening of skin (callus) at sites of abnormal load. Furthermore, ischaemic necrosis of tissues beneath the callus leads to breakdown of skin and subcutaneous tissue, resulting in a neuropathic ulcer. Autonomic neuropathy often results in changes to the texture and turgor of the skin, such as dryness and fissuring, creating a portal of entry for bacteria. Autosympathectomy with consequent sympathetic failure, arteriovenous shunting, and microvascular thermoregulatory disfunction impairs normal tissue perfusion and microvascular responses to injury. Another disorder contributing to the development of foot ulcers is peripheral vascular disease that affects the blood vessels of small and large sizes. Both macro- and microvascular diseases could contribute to the consequences of peripheral vascular disease, resulting in the inability of the dysvascular limb to heal itself properly. The incidence and prevalence of peripheral arterial disease (PAD) increase with age in both diabetic and nondiabetic subjects and, in those with diabetes, increase with the duration of diabetes. Hypertension, smoking, and hyperlipidemia, which are frequently present in patients with diabetes, contribute additional risk for vascular disease. Studies have shown that peripheral vascular disease develops at a younger age among patients with diabetes as compared to the general population [10]. Measurement of the ankle-brachial index (ABI), which represents the systolic blood pressure at posterior tibial or dorsalis pedis level compared with brachial blood pressure, can be used to define clinically occlusive PAD. In patients with an ABI < 0.90, the relative risk has been reported to be 1.25 (95% CI 1.05, 1.47) for developing an ulcer versus diabetic patients with a normal ABI [13]. Peripheral ischaemia resulting from proximal arterial disease was given as a component cause in the pathway to ulceration in 35% of cases in the two-centre study of causal pathways [14]. A recent comparative study of peripheral arterial disease in diabetic and nondiabetic patients confirmed that diabetic patients had more distal disease and a poorer outcome with respect to amputation and mortality [15]. The ischaemic foot is red, dry, and often neuropathic and it is also susceptible to pressure from, for example, footwear. Ulcerated diabetic foot is a complex problem and it is the result of the interaction of multiple causal factors such as neuropathy, peripheral vascular disease, trauma, and infections. Neuropathy and ischaemia are the initiating factors, most often together as neuroischaemia, whereas infection is mostly a consequence.

**Figure 1:**
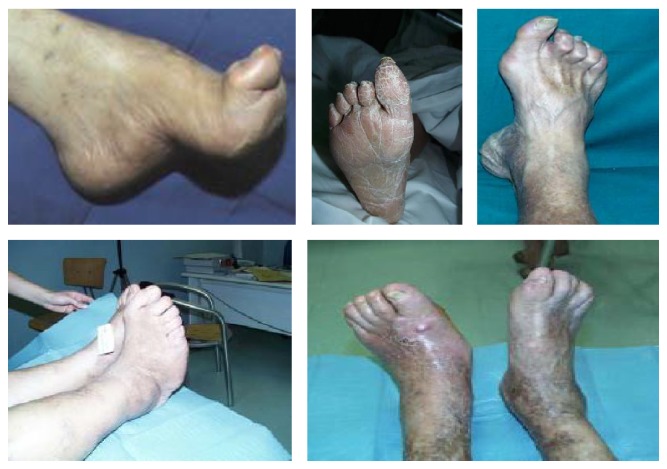
Neuropathic diabetic foot.

**Figure 2:**
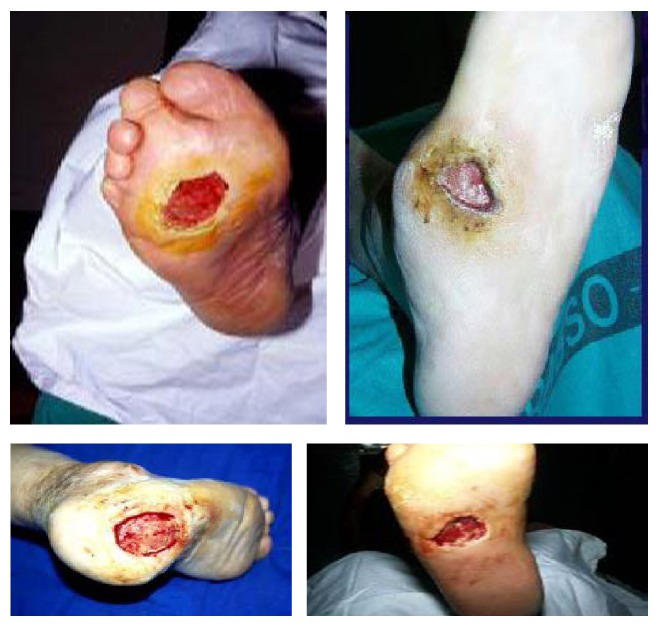
Neuropathic ulcers.

**Figure 3:**
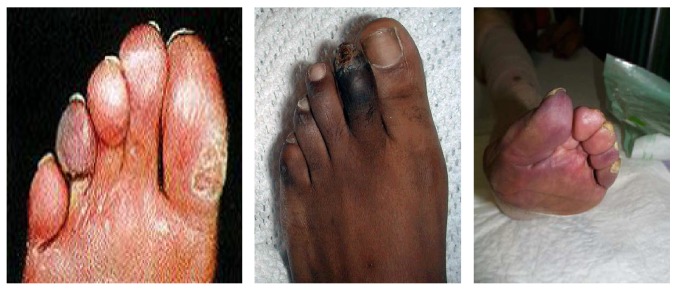
Ischemic diabetic foot.

**Figure 4:**
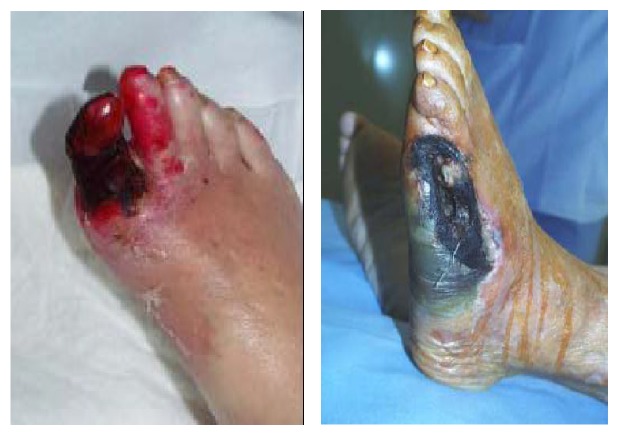
Infected diabetic foot.

**Figure 5:**
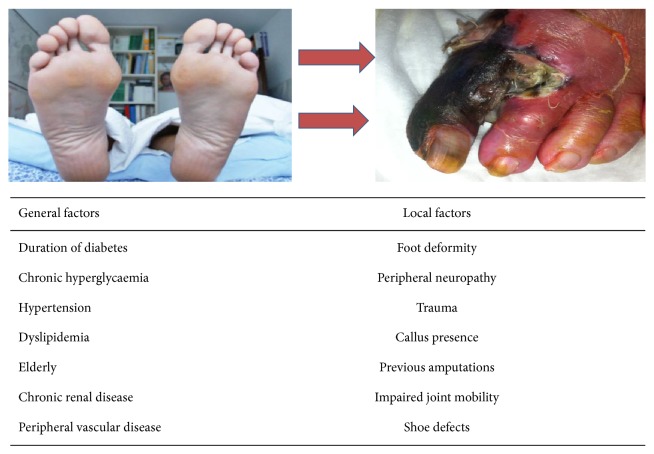
Evolving foot in diabetics: from healthy foot to ischemic diabetic foot.

**Figure 6:**
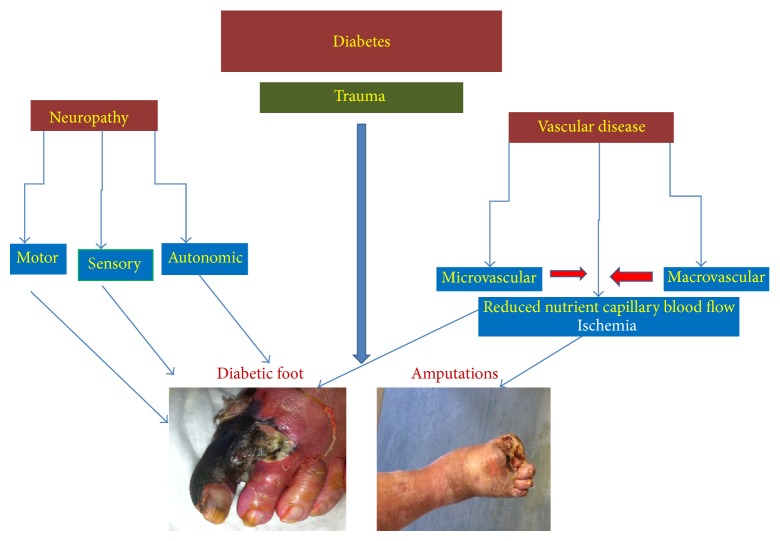
Pathogenetic pathway linking diabetes to diabetic foot ulceration.

It is possible to classify diabetic foot on a pathophysiological and clinical way in:* ischemic diabetic foot, neuropathic ischemic foot,* and* infected diabetic foot*, but this type of classification in clinical practice may appear too simple owing to the fact that it is possible to distinguish more frequent clinical variants with mixed features called* neuroischemic diabetic foot*. All these clinical variants of DFS have typical morphologic and clinical findings (see Figures 1, 2, 3, and 4).

In this review, we will reexamine some aspects of immune-inflammatory markers involvement in diabetic foot syndrome and the role of DFS as a possible marker of cardiovascular risk in diabetic subjects.

## 3. Cardiovascular Morbidity and Mortality of Disease in Diabetic Patients with DFS

Diabetic patients have a higher mortality compared with patients without diabetes. Several studies have reported that the rates of mortality and morbidity of CVD are 2–4 times higher among patients with type 2 diabetes mellitus than in nondiabetic subjects. Different studies also indicate that foot ulcers in diabetic patients are related to a higher mortality. In fact, diabetic foot is a major cause of morbidity in diabetic patients, and the mortality rate is about twice that of patients without foot ulceration [14, 16].

In a study [17] conducted by Pinto et al., these authors hypothesized that patients with type 2 diabetes mellitus with diabetic foot could have a worse prognosis in terms of faster progression of cardiovascular damage and higher cardiovascular morbidity. For this purpose, authors evaluated differences between subjects with type 2 diabetes mellitus with and without diabetic foot in the following: (1) cardiovascular risk profile, (2) cardiovascular morbidity prevalence by a retrospective evaluation, (3) prevalence of markers of subclinical cardiovascular damage at the time of recruitment, and (4) incidence of new-onset vascular events on a prospective analysis. They showed a higher prevalence of major cardiovascular risk factor, of subclinical markers of CVD, and of previous and new-onset cardiovascular and cerebrovascular events in diabetic patients with foot complications. These results could explain previous reports of high morbidity and mortality rates in diabetic patients with amputations [18–20]. The main cause of death in patients with diabetes was coronary artery disease (CAD) [18–20]. In this study, authors also reported a higher prevalence of major cardiovascular risk factors such as hypercholesterolemia, LDL plasma levels > 130 mg/dL, hypertriglyceridemia, and microalbuminuria/proteinuria in diabetic foot patients compared with diabetic patients without foot complications and this finding is consistent with the hypothesis that diabetic foot syndrome in diabetic subjects could represent a possible marker of cardiovascular risk. They also reported that patients with diabetic foot were more likely to have a cerebrovascular event (TIA and ischemic stroke) both on a retrospective evaluation (previous TIA and ischemic stroke) and on a prospective evaluation (new onset TIA and stroke on a 5-year follow-up). The most prevalent subtypes of stroke were lacunar and LAAS subtype with a slight higher prevalence of the lacunar subtype and this finding could suggest a role of both microvascular disease and atherosclerotic macroangiopathies, a determinant of vascular morbidity in patients with diabetic foot. The higher cardiovascular risk associated with diabetic foot may be related to a cumulative effect of the single risk factor linked to neuropathy and PAD, which are two well-known medical conditions recently associated with increased cardiovascular morbidity [18, 19], but another explanation could be recognized in the role of microangiopathy as a determinant of overall vascular risk. Nevertheless, because the groups evaluated in this study did not start with balanced CV risk factors and CV disease, authors cannot exactly correct for so many other powerful indicators especially with small numbers and relatively few events; so our findings can only warn clinicians that diabetic foot has to trigger a vital search for treatable cardiovascular risk factors and diseases.

The diabetic foot syndrome is the most frequent cause of hospitalization of diabetic patients and one of the economically most demanding complications of diabetes. People with diabetes have been shown to have higher mortality than people without diabetes, but the cerebrovascular risk profile of these patients is not fully evaluated. Another study [21] has been conducted to evaluate the possible role of diabetic foot as a cerebrovascular risk marker in type 2 diabetic patients. Authors enrolled 102 type 2 diabetes patients with diabetic foot and 123 diabetic patients without diabetic foot. Statistically significant differences were found in the distribution of the main cardiovascular risk factors with exception of hypertension. They observed a higher prevalence of previous cerebrovascular events (transient ischemic attack, ischemic stroke) and of incidence of new onset cerebrovascular events at a 5-year follow-up. Regarding clinical subtype of ischemic stroke classified according to Trial of ORG 10172 in Acute Stroke Treatment (TOAST) classification on a retrospective and prospective basis, we observed a higher prevalence of both the lacunar and large artery atherosclerosis subtype with a slight higher prevalence of lacunar subtype in patients with diabetic foot. These findings showed a worse cerebrovascular risk profile in diabetic patients with diabetic foot than in diabetic subjects without foot ulceration with a higher prevalence of cardiovascular risk factors and of anamnestic cerebrovascular events and incidence of new cerebrovascular events at a 5-year follow-up.

## 4. Cardiovascular Risk Factors in Diabetic Patients

### 4.1. Microalbuminuria

Microalbuminuria is defined by the detection of urinary albumin excretion rates of 30 to 300 mg in a 24 h urine collection. It is still the only anomaly of early diabetic kidney that has prognostic value statements. In fact, the appearance of microalbuminuria in diabetic patients is a very important index for progression to the most serious kidney disease. It represents a cardiovascular risk indicator in diabetic populations and also in hypertensive and general populations. Several studies and experimental data show in fact that microalbuminuria is associated with an increased risk for all-cause and cardiovascular mortality, cardiac abnormalities, cerebrovascular disease, and possibly PAD. In a recent study conducted by our group [22], we have showed the prevalence of microalbuminuria in patients with diabetic foot was higher than in patients without diabetic foot. In addition, we also showed a significant positive correlation between some clinical and laboratory variables, including microalbuminuria and the levels of interleukin 6 (IL-6) and resistin which are adipocytokines that may contribute to insulin resistance and to the development of inflammatory responses.

### 4.2. Hypertension

Hypertension is defined, according to the 1993 World Health Organization criteria, like a systolic blood pressure 140 mm Hg and/or diastolic blood pressure 90 mm Hg in subjects who are not taking antihypertensive medication. Diabetes mellitus and hypertension are both common diseases and they represent two powerful independent risk factors for cardiovascular, renal, and atherosclerotic disease. The pathogenesis of hypertension in diabetes type 1 and type 2 is different. Diabetic nephropathy is considered to be the main factor that contributes to the development of hypertension in patients with diabetes mellitus type 1. In the case of type 2 diabetes mellitus, hypertension is more often essential and it is part of a plurimetabolic syndrome in a context of insulin resistance. In all cases, hypertension worsens the prognosis of patients, increasing the risk of both macrovascular and microvascular complications. In fact, in the framework of diabetes and hypertension accelerates the development of diabetic retinopathy, nephropathy, and peripheral vascular disease. In diabetic patients with hypertension, blood pressure lowering helps to reduce significantly the treatment of cardiovascular and renal events. Therefore, it is necessary and appropriate treatment of hypertension in diabetic patients, which should include nonpharmacological interventions, drug therapy, regular monitoring of blood pressure, and educational endeavors. A study conducted by Pinto et al. [17] showed that the prevalence of hypertension was similar in both groups of diabetic patients with and without diabetic foot. In addition, we also showed a significant positive correlation between some clinical and laboratory variables, including hypertension and the levels of IL-6 and resistin which are adipocytokines that may contribute to insulin resistance and to the development of inflammatory responses.

### 4.3. Dyslipidemia

There are numerous cardiovascular diseases that occur in patients with diabetes, both type 1 or type 2. Dyslipidemia is one of the major risk factors for cardiovascular disease in diabetes mellitus. The defects in the synthesis and clearance of plasma lipoproteins are among the most commonly metabolic abnormalities that accompany diabetes. The diabetic dyslipidemia, a characteristic pattern characterized by the presence of low levels of high-density lipoprotein (HDL) cholesterol, hypertriglyceridemia, and postprandial lipemia, which is observed more frequently in type 2 diabetes, is one of several factors that contribute to accelerating macrovascular disease in diabetic patients. Among the different factors involved in developing of diabetic dyslipidemia insulin effects on liver apoprotein production, regulation of lipoprotein lipase (LpL), actions of cholesteryl ester transfer protein (CETP), and peripheral actions of insulin on adipose and muscle should be considered. The acknowledgment and treatment of dyslipidemia are therefore two important elements in the framework of a multidisciplinary approach aimed at the prevention of coronary heart disease. According to current guidelines for the prevention of coronary heart disease in diabetic patients elevated, LDL-C is the primary target of lipid-lowering therapy and statins are recommend as the first-line treatment for diabetic dyslipidemia. However, considering the complexity of the profiles of dyslipidemia in diabetic patients, multiple drugs are often required to achieve therapeutic targets. In addition, the other risk factors usually associated with diabetes mellitus, such as hypertension, hyperglycemia, and obesity, should be effectively managed to reinforce the effects of lipid-lowering therapy. In our recent study, we [17] showed a higher prevalence of dyslipidemia in patients with diabetic foot ulcers than in those without diabetic foot. In addition, we also showed a significant positive correlation between some clinical and laboratory variables, including dyslipidemia and the levels of IL-6 and resistin which are adipocytokines that may contribute to insulin resistance and to the development of inflammatory responses.

## 5. Inflammation Markers and Their Role in Cardiovascular Morbidity in DFS

In diabetes, there is a complex interplay of several variables with inflammatory metabolic disorders and their effect on the cardiovascular system. Simplified explanation may be that inflammation increases insulin resistance, which in turn leads to obesity, while perpetuates diabetes, high blood pressure, prothrombotic state, and dyslipidemia [20]. Some studies [23–25] suggest an interaction between hormones, cytokines, and resistin. Nevertheless, the circulating levels of adiponectin, which is the most abundant adipocytokine, are reduced in conditions such as obesity, type 2 diabetes, and coronary heart disease (CHD) [26–28]. In this context, hypoadiponectinemia was associated with low HDL-cholesterol (HDL-C) concentrations [26], decreased LDL particle size [27], and increased markers of systemic inflammation [29]. Jeffcoate et al. [30] suggested that in the diabetic foot there is an inflammatory cascade through an increased expression of proinflammatory cytokines, including tumor necrosis factor alpha (TNF-a) and interleukin-1b. Therefore, it is very suggestive that diabetic foot is characterized by a pronounced inflammatory reaction. Subclinical inflammation represents a risk factor for both type 2 diabetes and several diabetes-related complications, but data on diabetic neuropathies are scarce. Thus, some authors [31] investigated whether circulating concentrations of acute-phase proteins, cytokines, and chemokines differ among diabetic patients with or without diabetic polyneuropathy. They measured 10 markers of subclinical inflammation in 227 type 2 diabetic patients with diabetic polyneuropathy who participated in the population-based MONICA/KORA Survey F3. Diabetic polyneuropathy was diagnosed using the Michigan Neuropathy Screening Instrument (MNSI). After adjustment for multiple confounders, high levels of C-reactive protein and IL-6 were most consistently associated with diabetic polyneuropathy, high MNSI score, and specific neuropathic deficits, whereas some inverse associations were seen for IL-18. This study shows that subclinical inflammation is associated with diabetic polyneuropathy and neuropathic impairments and this association appears rather specific because only certain immune mediators and neuropathic impairments are involved.

Nevertheless, few data exist on the role of systemic inflammation in patients with diabetic foot syndrome although a low-grade immune activation represents an important risk factor not only for the development of type 2 diabetes but also for several vascular complications of diabetes such as macrovascular (myocardial infarction and stroke) and microvascular ones (neuropathy and nephropathy). The status of the immune system may be relevant at several stages in the development of chronic wounds. Immune activation may precede the incidence of a diabetic foot ulcer in the same way that it precedes the manifestation of type 2 diabetes and CHD. Since pro- and anti-inflammatory processes are crucial in the different phases of wound healing, it is well understood how the disturbances of the immune system interfere with tissue homeostasis and wound healing after the manifestation of ulcers leading to the chronic, nonhealing wounds that are characteristic of DFS.

Weigelt et al. [32] evaluated the association between foot ulcers and immune status in a cross-sectional study in diabetic patients with and without foot ulcers by measuring a range of immune mediators (acute-phase proteins, cytokines, and chemokines) representing different aspects of the immune system. These authors conducted this study to compare circulating levels of these immune mediators between both groups, to use multivariate regression models to identify potential confounders of these associations, and to investigate whether systemic immune activation was associated with the severity of the foot ulcer. Circulating levels of acute-phase proteins, cytokines, and chemokines were measured in diabetic patients with an ulcer and without an ulcer. Patients with an acute foot ulcer had higher levels of C-reactive protein (CRP), fibrinogen, IL-6, macrophage migration inhibitory factor, macrophage inflammatory protein-1*β*, and interferon-*γ*-inducible protein-10 as well as lower levels of RANTES (regulated on activation normal T-cell expressed and secreted), whereas no differences were found for IL-8, IL-18, and monocyte chemoattractant protein-1. In multivariate models, size of ulcer, according to the University of Texas classification, but not the grade of infection, was independently associated with three markers of subclinical inflammation (CRP, IL-6, and fibrinogen).

However, no study, to our knowledge, has evaluated the role of adiponectin, resistin, and immune-inflammatory biomarkers such as proinflammatory cytokines in patients with diabetic foot compared with those without foot complications.

On this basis, Tuttolomondo et al. conducted a study [22] with the aim to evaluate plasma levels of adiponectin, resistin, and IL-6 in subjects with diabetic foot in comparison with subjects without foot complications. Authors recruited 34 subjects with type 2 diabetes mellitus and foot ulceration hospitalized for every condition related to diabetic disease, but not for new vascular events (group A). As for controls, 37 patients with type 2 diabetes mellitus without foot ulceration have been recruited (group B), all hospitalized for every condition related to diabetic disease but not for new vascular events. Adiponectin, resistin, and IL-6 serum levels were evaluated. This study demonstrated that diabetic subjects with diabetic foot showed in comparison with diabetics without diabetic foot higher IL-6 and resistin plasma levels and lower adiponectin plasma levels. Resistin, although postulated to contribute to insulin resistance, may also contribute to inflammatory responses. Early investigations into the role of resistin as an inflammatory factor demonstrated that LPS (lipopolysaccharide) upregulated resistin expression in rat WAT, 3T3-L1 adipocytes, and human monocytes [33]. Although some initial studies in animal models have produced discrepancies about the fact that proinflammatory cytokines may regulate resistin, several recent human studies have supported the concept of inflammatory cytokine mediation of resistin [34, 35]. Moreover, Osawa et al. [36] reported that elevated serum resistin concentration appears to be an independent risk factor for ischemic stroke, especially lacunar and atherothrombotic infarction in the general Japanese population. In particular, in this study, authors showed that the combination of high resistin and the presence of either diabetes or hypertension increased the risk of ischemic stroke. This is consistent with our findings regarding the higher plasma levels of both IL-6 and resistin in diabetic subjects with foot ulceration in comparison with diabetics without foot complications. A recent study by Reilly and coworkers [37] suggested resistin as a metabolic link between inflammation and atherosclerosis. In contrast with resistin, adiponectin that is known to enhance insulin sensitivity and reduce atherosclerotic plaques, suppressed a resistin-mediated rise in VCAM-1 and ICAM-1 [38]. Adiponectin levels can be assessed by either of three variables: total adiponectin, HMWA, and the SA index.

Recently, Almeda-Valdes et al. [39] showed that total adiponectin, HMWA, and the SA index had similar utility for the identification of the metabolic abnormalities. This finding may stimulate the use of adiponectin in clinical and epidemiological settings because the measurement of total adiponectin is better standardized, cheaper, and more accessible than the other two approaches. Hypoadiponectinemia can be viewed as an early sign of a complex cardiovascular risk factor predisposing to the atherosclerosis process as well as a contributing factor accelerating the progress of the atherosclerotic plaque. Adiponectin also exhibits anti-inflammatory and atheroprotective actions in various tissues by suppressing the expression of vascular adhesion molecules and scavenger receptors, reducing the expression of the inflammatory cytokine TNF-a, raising NO production, and suppressing the proliferation and migration of smooth muscle cells [40]. Furthermore, two receptors that mediate adiponectin's actions in fatty-acid oxidation and glucose uptake, namely, ADIPOR1 and ADIPOR2 [41], have been identified. Very recently, Halvatsiotis et al. [42] have demonstrated for the first time that a sequence variant in the intron 5 of the ADIPOR2, rs767870 among the eight studied, is associated with cardiovascular disease in a population of Greek individuals. Recent findings by Tuttolomondo et al. [22] of lower median plasma levels of adiponectin in subjects with diabetic foot could confirm this issue. Furthermore, the same authors observed a significant negative correlation between adiponectin plasma levels and some cardiovascular risk factors such as hypertension, dyslipidemia, and clinical variables indicating previous cardiovascular morbidity such as previous TIA/stroke and incident vascular morbidity such as neuropathy, microalbuminuria, and PAD and these findings further suggest a possible role of hypoadiponectinemia as a putative marker of cardiovascular morbidity both prevalent and incident. As several cytokines are also produced by adipose tissue [43], it was postulated that an “adipovascular” axis [44] may contribute to the increased risk of cardiovascular events in patients with type 2 diabetes. In patients with diabetic foot, this “adipovascular axis” expression in lower plasma levels of adiponectin and higher plasma levels of IL-6 could be linked to foot ulcers pathogenesis by microvascular and inflammatory mechanisms. Furthermore, obesity correlates with metabolic complications of obesity. Some authors [45] evaluated adipocyte volume and its relationship with TNF-a, IL-6, adiponectin, and high-sensitivity C-reactive protein (hs-CRP) levels. Patients were divided into 4 groups: lean healthy controls [body mass index (BMI): 24.2 ± 1.4 kg/m^2^], nondiabetic obese patients (30.2 ± 2.9), obese (30.1 ± 3.2), and nonobese (22.2 ± 1.5) type 2 diabetic patients. TNF-a, hs-CRP, adiponectin, and IL-6 levels were measured preoperatively and SC fat specimens were obtained during operation. In this study, mean adipocyte volumes were higher in obese diabetic patients than in other groups. Mean TNF-a, hs-CRP, and IL-6 levels were higher in obese diabetic patients than in control subjects, obese nondiabetic, and nonobese diabetic patients. Mean TNF-a levels of nondiabetic obese patients were higher than the control group. Mean IL-6 levels of diabetic and nondiabetic obese patients were higher than control subjects. Mean adiponectin levels of control subjects were higher than nondiabetic obese, nonobese diabetic, and obese-diabetic subjects. Mean adiponectin levels of obese diabetic patients were lower than nondiabetic obese subjects. Mean hs-CRP levels were higher in diabetic patients whether they were obese or not. Authors observed a positive correlation between adipocyte size and TNF-a, IL-6, and hs-CRP levels and negative correlation between adipocyte size and adiponectin levels. These findings furtherly confirm the existence of an adipose-inflammatory vascular axis strictly involved in diabetic complications such as DFS owing to the fact that adiposity and its related conditions, such as diabetes, are at the same time pro-inflammatory and inflammatory conditions.

Adipokines are markers of insulin resistance and play a role in the atherosclerotic process [44].

Indeed, recent studies suggest that adiponectin may play a role in the modulation of inflammatory vascular response by inhibiting the expression of adhesion molecules on endothelial cells [40], inhibiting endothelial cell NF-*κ*B signaling [46], and suppressing macrophage function [47, 48]. Other studies showed that adiponectin suppressed the TNF-a stimulated expression of E-selectin, VCAM-1, and ICAM-1 in human endothelial cells [40, 46]. This suggests further that adiponectin may be vasoprotective as reported by other authors [47, 48] and negatively modulate the atherogenic processes and that adiponectin has an interaction with an important inflammatory cytokine such as TNF-a.

To evaluate the association of adipokines with the macrovascular complications of type 1 diabetes mellitus, a study [47] has been conducted, evaluating serum adiponectin, leptin, and resistin levels in type 1 diabetic patients and analyzing their relationship with carotid intima media thickness (CIMT). The authors showed that adiponectin levels in diabetics were higher and leptin levels were lower than controls. Resistin levels were also higher in the diabetic group compared to controls. Adiponectin was also negatively correlated with CIMT, age, BMI, and waist-to-hip ratio and positively with creatinine. Leptin levels were correlated with total cholesterol and high-density lipoprotein (HDL). Resistin was correlated with CIMT and systolic blood pressure. Authors concluded that increased adiponectin correlates negatively and resistin positively with CIMT in type 1 diabetic patients, thus representing possible candidate markers of adipose-inflammatory dysfunction also in diabetic type 1 subjects and possible markers of cardiovascular risk.

The pathophysiology of insulin resistance and atherosclerosis share a common inflammatory basis. On this basis, some authors [48] to test this hypothesis evaluated 40 patients with a myocardial infarction (MI). Endothelium-dependent (FMD (flow-mediated dilation)) and independent (NTG (nitroglycerine)) vasodilatation (determined by ultrasound), S(I) (insulin sensitivity index; determined by isoglycemic-hyperinsulinaemic clamp) and serum levels of CRP (C-reactive protein), TNF-a, IL-6, resistin, and adiponectin (determined by ELISA) were measured. FMD, S(I), and adiponectin levels resulted significantly lower in patients with type 2 diabetes mellitus, whereas TNF-a and IL-6 levels have been observed as significantly higher in the same diabetic patients. Authors also reported that TNF-a concentrations and brachial artery diameter were negatively correlated with FMD whereas S(I) was positively correlated with FMD. These results indicate how endothelium is negatively impacted in multiple ways by the diabetic state after an MI also suggesting endothelium as the main organ-target of adipose-inflammatory dysfunction of diabetes.

Recently, Zietz et al. [46] reported that low levels of adiponectin are associated with low levels of HDL-cholesterol and might represent an independent cardiovascular risk factor, whereas high levels of adiponectin are associated with high levels of HDL-cholesterol indicating a protective risk profile. Furthermore, Tuttolomondo et al. [22] observed significant either positive (for IL-6 and resistin) and negative (for adiponectin) correlations in subjects with diabetic foot between these immunoinflammatory and metabolic markers and some clinical and laboratory variables and these correlation further underline the relationships with inflammatory background. Recently, Pinto et al. underlined [17] the role of DFS to predict cardiovascular morbidity in diabetic patients, even after correction for other well-known cardiovascular risk factors. In our study, both univariate and multivariate analyses showed the predictive positive role of resistin and IL-6 plasma levels and a negative one of adiponectin towards diabetic foot presence. Previous studies have shown the relationship between inflammatory cytokines and cardiovascular morbidity in diabetic patients. Tuttle et al. [49] showed that both IL-6 and TNF-a are chronically increased in diabetic women with and without CVD compared to nondiabetic women. The additive concentration of cytokines in diabetes and CVD suggests a common inflammatory state in both diabetes and CVD.

Thus, most recent evidences suggest that diabetic atherosclerosis is not only a disease of hyperlipidemia simply, but is also an inflammatory disorder. A study [50] has been conducted to evaluate the prevalence of inflammatory markers such as high-sensitivity C-reactive protein (hsCRP), adiponectin, and nuclear factor-*κ*B (NF-*κ*B) expression, in peripheral blood mononuclear cells in Indian patients with type 2 diabetes mellitus with and without macrovascular disease. Authors enrolled 29 consecutive cases of type 2 diabetic patients with proven MVD (group A), 28 matched cases without MVD (group B), and 14 healthy controls (group C) were evaluated for the clinical parameters fasting blood glucose (FBG), 2 h postprandial blood glucose (PPBG), glycosylated hemoglobin (HbA1c), lipid profile, and the above-mentioned inflammatory markers.

The authors reported that subjects with type 2 diabetes showed higher hs-CRP and NF-*κ*B expression and lower values of adiponectin compared to healthy controls. Group A had significantly higher serum hs-CRP than group B (*P* = 0.0001) despite comparable values of BMI, FBG, 2-h PPBG, HbA1c, and lipid parameters. Group A had significantly higher serum hs-CRP and NF-*κ*B expression and significantly lower levels of adiponectin than group C. In Group A, serum adiponectin negatively correlated with NF-*κ*B expression. In Group B, adiponectin values correlated negatively with both FBG and 2-h PPBG. These finding further suggest that patients with type 2 diabetes were in a proinflammatory state strictly related to subsequent vascular complications such as diabetic foot syndrome.

## 6. Discussion

Several diabetic foot problems including ulcerations, infections, and gangrene represent the most common cause of hospitalization among diabetic patients and their management, cost millions of euro every year, and place a tremendous burden on the health care system. Peripheral sensory neuropathy, deformity, and trauma are the most common features underlying diabetic foot ulcers although other risk factors such as calluses, edema, and peripheral vascular disease have also been identified as etiological factors contributing to the development of diabetic foot ulcers. Although the pathogenesis of peripheral sensory neuropathy is still poorly understood, there seem to be multiple mechanisms involved, including the formation of advanced glycosylated end products and diacylglycerol, oxidative stress, and activation of protein kinase C*β*. The data linking glycemic control and neuropathy are not as clear cut as those for retinopathy because of the difficulty in identifying objective measures to assess the many stages of neuropathy over time and because the symptoms, or lack thereof, of neuropathy may be misleading if assessed only through patient questionnaires. Finally, the differential diagnosis of peripheral neuropathy is quite large, and patients may have other etiologies as well. The diabetic foot can be classified into the neuropathic foot, characterized by the neuropathic ulcer, the Charcot joint, and neuropathic oedema associated with a good circulation, in which neuropathy predominates, and the ischaemic foot in which atherosclerosis is the dominant factor leading to a reduction in blood flow with absent pulses. In the neuropathic foot, blood flow is increased, the vessels are still and dilated as a result of medial wall calcification and there is evidence for arteriovenous shunting. The neuropathic ulcer characteristically develops on the plantar surface following inflammatory autolysis and haematoma formation under neglected callosities. Chiropody is therefore the mainstay of treatment and recurrence is prevented by redistribution of weight bearing forces by moulded insoles in special footwear. Charcot osteoarthropathy is often preceded by fracture which is a further a complication of diabetic neuropathy and precipitates the rapid bone and joint destruction of the Charcot joint. The ischaemic foot is characterized by rest pain, ulceration, and gangrene. Medical management can be successful in up to 72%, with the remainder needing arteriography to assess suitability for arterial reconstruction or angioplasty. In the diabetic leg, atherosclerosis is predominant in the branches of the popliteal artery making arterial reconstruction difficult. Diabetes and its vascular complication have also a clear inflammatory pathogenesis, but few studies evaluated immunoinflammatory background of diabetic foot syndrome (DFS). Only few previous studies [17, 21, 22] evaluated inflammatory markers such as cytokine and adipokines in patient with diabetic foot. Hypoadiponectinemia can be viewed as an early sign of a complex cardiovascular risk factor predisposing to the atherosclerosis process and a contributing factor accelerating the progress of the atherosclerotic plaque. Adiponectin exhibits anti-inflammatory and atheroprotective actions in various tissues by suppressing the expression of vascular adhesion molecules and scavenger receptors, reducing the expression of the inflammatory cytokine TNF-a, raising NO production, and suppressing the proliferation and migration of smooth muscle cells. These data are consistent with the findings reported by Tuttolomondo et al. [22] of lower median plasma levels of adiponectin in subjects with diabetic foot. Furthermore, we observed a significant negative correlation between adiponectin plasma levels and some cardiovascular risk factors such as hypertension, dyslipidemia, and clinical variables indicating previous cardiovascular morbidity such as previous TIA/Stroke and incident vascular morbidity such as neuropathy, microalbuminuria, and PAD and these findings further suggest a possible role of hypoadiponectinemia as a putative marker of cardiovascular morbidity both prevalent and incident. As several cytokines are also produced thus an “adipovascular” axis may contribute to the increased risk of cardiovascular events in patients with type 2 diabetes. In patients with diabetic foot this “adipovascular axis” expression in lower plasma levels of adiponectin and higher plasma levels of IL-6 could be linked to foot ulcers pathogenesis by microvascular and inflammatory mechanisms. These findings further underline the importance of inflammatory and metabolic “milieu” such as cytokines and adipose hormones in foot complications in diabetics as already reported for other vascular complications of diabetes [45, 51–61].
